# Supplementary material for: Prediction of Anti-Cancer Drug-Induced Pneumonia in Lung Cancer Patients: Novel High-Resolution Computed Tomography Fibrosis Scoring
Source: J Clin Med. 2020 Apr 7;9(4):1045. doi: 10.3390/jcm9041045 (PMC7230276; doi:10.3390/jcm9041045)
Supplement: Supplementary file 1 [file jcm-09-01045-s001.pdf]

Supplementary Information template:

Supplementary

TITLE: Prediction of anti-cancer drug-induced pneumonia in lung cancer patients: Novel high-resolution computed tomography fibrosis scoring

Supplementary Table S1. List of anti-cancer drug induced pneumonia patients (*n* = 19).

HFS	Therapy †	Anti-cancer drug regimen						Grade ‡
		1st	2nd	3rd	4th	5th	6th	
122.5	O→CR→C	CDDP+S1	DTX§					3
122.5	C	CBDCA+PTX	S1§					1
115.5	C	CDDP+Gem	S1§	DTX				1
112	C	CBDCA+PTX	Pem§	S1§				1, 1
108	CR	CBDCA+nabPTX *						5
107.5	C→R→C	CDDP+Pem+Bev *	CBDCA+PTX+Bev	nabPTX §				2, 5
107.5	C	CDDP+Pem §						3
107.5	CR	CDDP+AMR §						2
107	C	CBDCA+Pem §						1
104	O→R→C	UFT	CBDCA+PTX+Bev	Pem	DTX §			2
104	R→C	CBDCA+ETP	ETP	CPT-1 1§				2
102.5	CR→O	CDDP+S1	Pem§					2
102.5	C	Gefitinib	ETP§					5
102	C	Gefitinib §						2
102	CR	CDDP+S1 §						2
100	C	Gefitinib	CBDCA+PTX+Bev	Pem	Afatinib	DTX	Erlotinib §	3
100	O→R→C	Gefitinib §						2
100	O→R→C	CBDCA+PTX	CDDP+Gem	DTX	Elrotinib §			1
100	C	Erlotinib	CBDCA+Pem §					1

† Sequence of anti-cancer therapies. C, chemotherapy; R, radiation; O, operation; CR, chemo-radiation. ‡ CTCAE version 4.0. Two patients developed anti-cancer drug induced pneumonia twice.§ Suspected drugs CDDP, cisplatin; S1, TS-1® (Thaiho Pharmaceutical Co.Ltd, Tokyo, Japan); DTX, docetaxel; CBDCA, carboplatin; PTX, paclitaxel; Gem, gemcitabine; Pem, pemetrexed; nabPTX, nanoparticle albumin-bound paclitaxel; Bev, bevacizumab; AMR, amrubicin, UFT, tegafur-uracil; ETP, etoposide; CPT-11, irinotecan.
